# RhoA: A therapeutic target for chronic myeloid leukemia

**DOI:** 10.1186/1476-4598-11-16

**Published:** 2012-03-25

**Authors:** Poonam R Molli, Madhura B Pradhan, Suresh H Advani, Nishigandha R Naik

**Affiliations:** 1 Cancer Research Institute, ACTREC, Tata Memorial Centre, Kharghar, Navi Mumbai 410210, India; 2 Department of Medical Oncology, Tata Memorial Hospital, Tata Memorial Centre, Parel, Mumbai 400012, India; 3 Center for Advanced Biotechnology and Medicine (CABM), University of Medicine and Dentistry (UMDNJ), Robert Wood Johnson Medical School, Piscataway, New Jersey 08854, USA; 4 Imaging and Cancer Biology, Piramal Healthcare Ltd, 1 Nirlon Complex, Off Western Express Highway, Mumbai 400063, India; 5 Director, Department of Medical Oncology, Jaslok Hospital and Research Centre, 15 Dr. Deshmukh Marg, Mumbai 400026, India

**Keywords:** Chronic Myeloid Leukemia (CML), Actin, RhoGTPases, Polymorphonuclear leukocytes (PMNL), n-formyl-methionyl-leucyl-phenylalanine (fMLP), Signal transduction

## Abstract

**Background:**

Chronic Myeloid Leukemia (CML) is a malignant pluripotent stem cells disorder of myeloid cells. In CML patients, polymorphonuclear leukocytes (PMNL) the terminally differentiated cells of myeloid series exhibit defects in several actin dependent functions such as adhesion, motility, chemotaxis, agglutination, phagocytosis and microbicidal activities. A definite and global abnormality was observed in stimulation of actin polymerization in CML PMNL. Signalling molecules ras and rhoGTPases regulate spatial and temporal polymerization of actin and thus, a broad range of physiological processes. Therefore, status of these GTPases as well as actin was studied in resting and fMLP stimulated normal and CML PMNL.

**Methods:**

To study expression of GTPases and actin, Western blotting and flow cytometry analysis were done, while spatial expression and colocalization of these proteins were studied by using laser confocal microscopy. To study effect of inhibitors on cell proliferation CCK-8 assay was done. Significance of differences in expression of proteins within the samples and between normal and CML was tested by using Wilcoxon signed rank test and Mann-Whitney test, respectively. Bivariate and partial correlation analyses were done to study relationship between all the parameters.

**Results:**

In CML PMNL, actin expression and its architecture were altered and stimulation of actin polymerization was absent. Differences were also observed in expression, organization or stimulation of all the three GTPases in normal and CML PMNL. In normal PMNL, ras was the critical GTPase regulating expression of rhoGTPases and actin and actin polymerization. But in CML PMNL, rhoA took a central place. In accordance with these, treatment with rho/ROCK pathway inhibitors resulted in specific growth inhibition of CML cell lines.

**Conclusions:**

RhoA has emerged as the key molecule responsible for functional defects in CML PMNL and therefore can be used as a therapeutic target in CML.

## Background

Chronic myeloid leukemia (CML) is characterized by the presence of Philadelphia (Ph^1^) chromosome bearing chimeric bcr-abl gene that translates a protein p210 which has increased and unregulated tyrosine kinase activity [1]. Polymorphonuclear leukocytes (PMNL) are terminally differentiated myeloid cells that play a crucial role in host defence by migrating to the sites of infection and eliminating foreign bodies. This complex process involves a cascade of signalling events that results in sequential stimulation of chemotaxis, phagocytosis, degranulation and oxidative burst. PMNL from CML patients exhibit defects in several actin dependent functions such as motility, chemotaxis, adhesion, aggregation, endocytosis, microbicidal activities and polymerization of actin per se [2]. Bcr-abl has an actin-binding domain that enhances its transforming ability. Targets of bcr-abl are similar to the major components of signal transduction pathways leading to actin polymerization. These include ras, PI3K, MAPK, JNK/SAPK, NF-kB and STAT. Ras and other oncoproteins require active rhoGTPases to elicit their transforming activities [3]. RhoGTPases also regulate spatial localization of F-actin. Since ras and rhoGTPases play significant role in actin polymerization and cell transformation, to understand their role in the pathogenesis of CML, the present study is focused on the status of these GTPases and actin in normal and CML PMNL. The results suggest a significant role of rhoA in functional defects of CML PMNL and identify rhoA as a therapeutic target in CML.

## Results

A classical chemoattractant - n-formyl-methoinyl-leucyl-phenyl alanine (fMLP) binds to its receptors on PMNL and initiates a cascade of signalling pathways that leads to various morphological, biochemical and functional events. On exposure to fMLP, PMNL show polarization [4]. Polarization of PMNL is associated with polymerization of actin that occurs in two phases - rapid rise in F-actin that peaks around 10-15 sec and decays after a half time of 30 sec and a second phase which decays after about 3 min. Various actin dependent events such as release of Ca^+2^, cell polarization, cell motility and chemotaxis are initiated in the first phase, while phagocytosis and oxidative burst are observed later. Therefore, polymerization of actin and status of rhoGTPases were studied after fMLP stimulation, at early time points - 0.5 and 5 min and later time points - 10, 30, 45 and 60 min.

### CML PMNL do not show classical morphological responses

**Figure 1:**
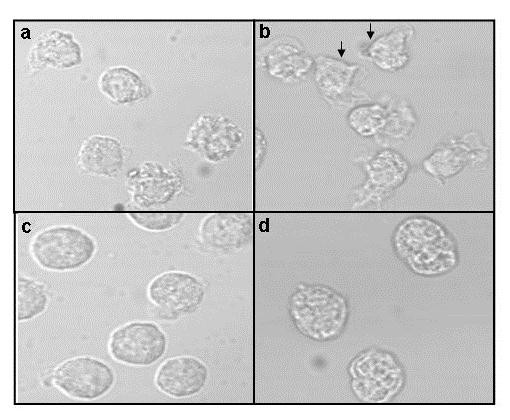
**Morphology of PMNL**: Phase contrast micrographs showing morphology of **a**) unstimulated and **b**) fMLP stimulated (0.5 min) normal PMNL. Arrows indicate classical morphology of oriented cells showing lamellipodia and uropods. **c**) Unstimulated and **d**) fMLP stimulated (0.5 min) CML PMNL.

Unstimulated normal PMNL were round (Figure 1a). After fMLP stimulation for 0.5 min, 90% of PMNL showed either blebbing or classical oriented cells with lamellipodia and uropod (Figure 1b). At 5 min, the cells became elongated and later they rounded up. Unstimulated CML PMNL were round (Figure 1c). At early time points of fMLP stimulation, in about 45% of samples, 50% cells showed fine peripheral projections (Figure 1d). Classical lamellipodia and uropod formation was rare. With increasing time the cells rounded up. Thus, CML PMNL exhibited lack of morphological responses towards fMLP.

### Actin expression is lower and stimulation of actin polymerization is absent in CML PMNL

**Figure 2:**
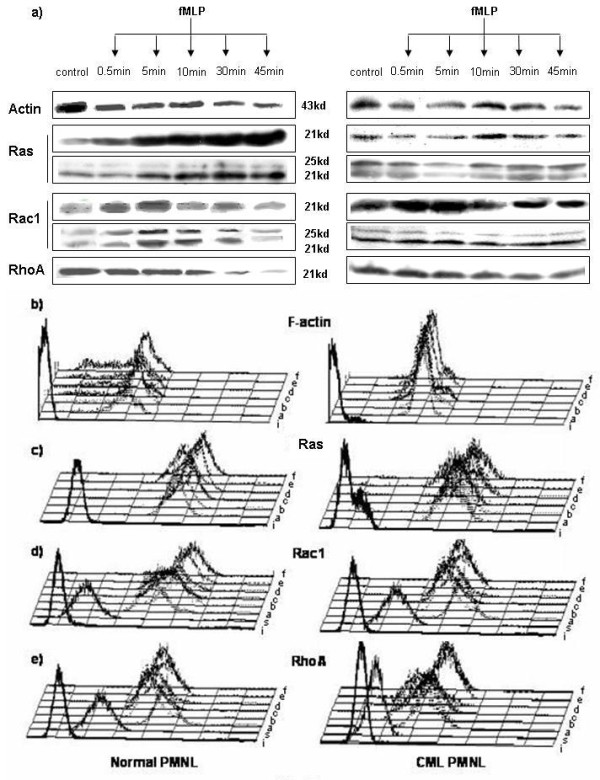
**Time kinetics of GTPase and actin stimulation**: (**a**) Western blot for expression of actin and GTPases - ras, rac1 and rhoA: lane 1- unstimulated control, lane 2- fMLP 0.5 min, lane 3- fMLP 5 min, lane 4- fMLP 10 min, lane 5- fMLP 30 min and lane 6- fMLP 45 min in normal (n = 14) and CML PMNL (n = 22); For ras and rac1 both the patterns of expression, i.e., 21 kd alone or 21 kd along with 25 kd are depicted. (**b-e**) Representative FCM plots for expression of F-actin and GTPases- Ras, rac1 and rhoA in normal (n = 14) and CML PMNL (n = 22): **i**) isotype, **s**) secondary control, **a**) unstimulated, **b**) fMLP 0.5 min, **c**) fMLP 5 min, **d**) fMLP 10 min, **e**) fMLP 30 min, and **f**) fMLP 45 min.

Western blot analysis would estimate expression of total actin i.e., G plus F actin in the cells. In normal PMNL, at early time points of fMLP stimulation, 60% of the normal samples showed a drop in total actin as compared to that in unstimulated PMNL (Figure 2a). The total actin reduced significantly by 20% and 36% after 5 and 45 min of fMLP stimulation, respectively (Table 1a). In CML PMNL, 50% of the samples showed a slight drop in total actin during early time points of fMLP stimulation (Figure 2a). The average total actin dropped significantly (15%) at 0.5 and 45 min of fMLP stimulation (Table 1a). Though unstimulated and fMLP-stimulated normal PMNL showed higher levels of total actin as compared to the levels in the respective CML PMNL, this difference was not statistically significant.

**Table 1:** Quantitation of GTPases and actin expression

a) Densitometric analysis of Western blots of GTPases - ras, rac1 and rhoA, and actin in normal and CML PMNL							
**Protein**	**PMNL**	**Unstimulated**	**fMLP stimulation**				
			
			**0.5 min**	**5 min**	**10 min**	**30 min**	**45 min**

**Actin**	**Normal**	52.89 ± 10.55	42.89 ± 9.01	42.22 ± 9.48 $	46.67 ± 8.90	42.44 ± 10.23	34.22 ± 8.61 $


	**CML**	39.50 ± 8.74	32.70 ± 7.58 $	35.80 ± 9.04	37.50 ± 8.44	32.30 ± 8.47	33.00 ± 9.09 $

**Ras**	**Normal**	37.80 ± 9.11	41.00 ± 8.05	46.71 ± 6.52	52.43 ± 9.61	58.29 ± 8.60 $	59.79 ± 10.66 $

	**CML**	37.37 ± 8.90	41.10 ± 12.65	46.31 ± 13.52	45.37 ± 12.60	46.04 ± 12.00 #	47.72 ± 13.93

**Rac 1**	**Normal**	58.22 ± 9.47	67.29 ± 9.30	70.64 ± 8.1	63.00 ± 9.58	60.21 ± 8.58	49.21 ± 7.44

	**CML**	63.05 ± 11.33	69.90 ± 9.11	81.17 ± 11.47 $	82.36 ± 11.21 $	75.77 ± 9.57 $	65.14 ± 9.77

**Rho A**	**Normal**	62.71 ± 12.45	65.14 ± 15.15	49.64 ± 10.88	43.36 ± 11.46 $	38.31 ± 10.79 $	24.75 ± 7.44

	**CML**	97.18 ± 14.24	94.27 ± 12.61	78.14 ± 11.09	78.19 ± 10.59 #	75.59 ± 9.99 #	78.00 ± 12.01 $#

b) Median fluorescence channels in flow cytometric estimation of GTPases - ras, rac1 and rhoA, and F-actin in normal and CML PMNL							

**Protein**	**PMNL**	**Unstimulated**	**fMLP stimulation**				
			
			**0.5 min**	**5 min**	**10 min**	**30 min**	**45 min**

**F-Actin**	**Normal**	28.53 ± 3.19	36.79 ± 2.43 $	32.64 ± 2.48	34.39 ± 2.06	30.52 ± 3.54	26.64 ± 3.44
	
	**CML**	22.75 ± 2.09	23.42 ± 2.29 #	20.58 ± 1.92 #	20.01 ± 1.89 #	23.97 ± 3.12	24.65 ± 3.34

**Ras**	**Normal**	122.21 ± 24.83	133.14 ± 17.94	180.49 ± 8.65 $	179.18 ± 4.86 $	169.19 ± 26.33	183.75 ± 27.48
	
	**CML**	99.86 ± 9.76	103.51 ± 13.76	102.38 ± 12.88#	117.40 ± 17.25 #	115.69 ± 15.40	126.65 ± 16.27 $

**Rac 1**	**Normal**	176.62 ± 20.81	225.95 ± 46.83	186.68 ± 30.36	194.79 ± 30.48	199.84 ± 29.24	217.03 ± 25.76
	
	**CML**	190.82 ± 19.64	118.94 ± 10.18 $	129.79 ± 13.71 $	132.73 ± 12.81 $	170.33 ± 22.15	158.16 ± 17.09 $#

**Rho A**	**Normal**	84.45 ± 11.65	82.77 ± 10.91	67.84 ± 6.92	72.79 ± 7.20	72.63 ± 10.34	65.71 ± 8.58
	
	**CML**	73.83 ± 11.33	60.68 ± 10.14	61.33 ± 10.95	62.74 ± 11.41	76.22 ± 13.57	76.33 ± 12.97

Values represent Mean ± S.E.M.; Normal - n = 14 and CML - n = 22; $ = significantly different as compared to the unstimulated control; # = significantly different as compared to the respective normal PMNL.

Flow cytometry (FCM) analysis showed that on fMLP stimulation for 0.5 min, F-actin in normal PMNL increased significantly (range 1.1 - 3.5 fold) (Figure 2b). This was followed by a drop and then a second increase. Since the time frame for this second response varied from sample to sample, the average of the median fluorescence did not show considerable change (Table 1b). In CML, only 25% samples showed an increase in F-actin after 0.5 min of stimulation that was comparable to that in normal PMNL (Figure 2b). Overall, F-actin levels were steady in fMLP stimulated CML PMNL (Table 1b). Thus, CML PMNL showed defective stimulation of actin polymerization.

### Organization of F-actin is altered in CML PMNL

**Figure 3:**
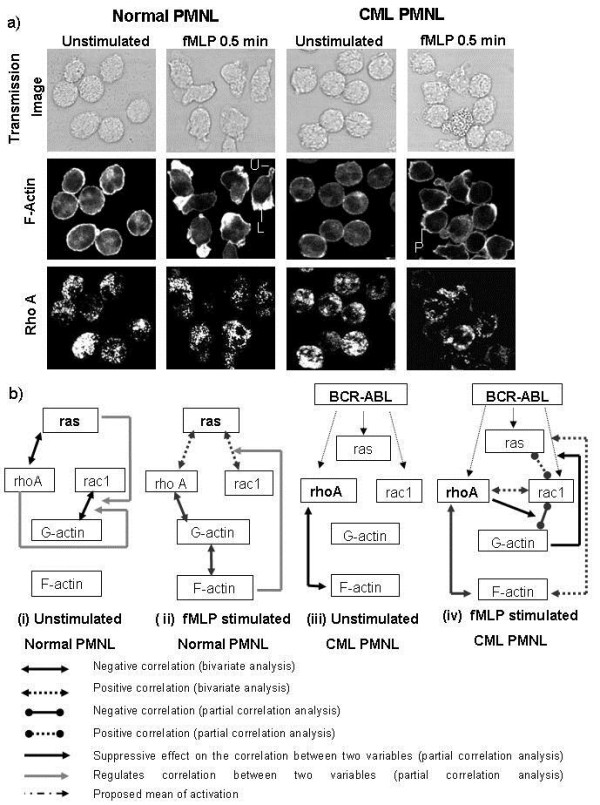
**(a) F-actin and rhoA localization**: Middle Z planes of LCM images showing transmission images (upper panel) and distribution of F-actin (middle panel) and rhoA (lower panel) in unstimulated normal and CML PMNL and those stimulated with fMLP (0.5 min); U - Uropod, L - Lamellipodia, P - Projection. **(b) **Proposed model for interactions between GTPases and F-actin: (i) Unstimulated and (ii) fMLP stimulated normal PMNL; (iii) unstimulated and (iv) fMLP stimulated CML PMNL. Bold font indicates crucial regulatory molecules.

Unstimulated normal PMNL showed F-actin as weak cytoplasmic and bright peripheral fluorescence. At 0.5 min of stimulation, an increase in F-actin along with cell polarization was seen. F-actin was concentrated in blebs or lamellipodia and uropods (Figure 3a). With increasing time, the pattern of F-actin distribution was similar to that seen in the unstimulated cells. Changes in F-actin levels observed by laser scanning confocal microscope (LCM) were similar to those seen using FCM. In unstimulated CML PMNL, F-actin was seen as weak cytoplasmic and slightly brighter peripheral fluorescence. After fMLP treatment, though morphological changes were observed in some cells, F-actin picture remained unaltered (Figure 3a).

Actin provides structural framework and defines cell shape and polarity. Its dynamic properties provide the driving force for the cells to move and divide. Changes in its expression could alter G to F polymerization dynamics. Defects in actin could be at the level of expression or polymerization. Alterations within actin could be via mutations in actin, changes in upstream regulatory signaling proteins or actin binding proteins. Altered actin expression and polymerization is known to be associated with cancer [5]. In normal and CML PMNL, basal levels of total actin and F-actin were not significantly different. On fMLP treatment, total actin levels decreased in both, but normal PMNL showed significant increase in F-actin, indicating that the total actin expression remained above the critical levels and hence actin could polymerize. Tarachandani et al have shown that in CML PMNL, actin expression, though lower, was sufficient for polymerization [6]. Hence, lack of stimulation of actin polymerization and altered F-actin architecture in CML PMNL could be due to defects in signalling leading to defective actin polymerization. The signalling molecules - rhoGTPases, play an important role in the spatial and temporal organization of actin. Ras, the major target of bcr-abl, activates rhoGTPases. In the present studies, on fMLP stimulation CML PMNL showed filopodia-like but thin projections, suggesting involvement of rhoA. This finding was different from that in normal PMNL where lamellipodia formation indicative of rac signaling were seen [7]. Since rhoA and rac govern dynamics and spatial organization of F-actin differently, changes in actin expression and F-actin localization, leading to defective functions [4,8] in CML PMNL, could be due to altered rhoGTPases regulation.

About 70% of the bcr-abl protein localizes to the cytoskeleton. This localization is important in cellular transformation. An actin-binding domain of the bcr-abl kinase enhances its leukemogenicity [9]. Analogously, a mutation in the F-actin binding domain of c-abl reduces its ability to transform fibroblasts [10]. Constitutively active bcr-abl alters actin dependent cell adhesion and motility by phosphorylating actin-binding proteins [11]. Another mechanism that alters actin functioning in the neoplastic state targets upstream regulators of actin binding proteins. Ras - the key signalling molecule in the actin polymerization pathway, is also a major target of bcr-abl. Ras regulates cell proliferation pathways that in turn regulate gene expression, and morphological (or motility) pathways controlling the actin cytoskeleton [12]. Therefore, the GTPases - ras, rho A and rac1 were studied.

### Ras expression in CML PMNL is refractory to fMLP treatment

In the majority of normal and CML PMNL, a sharp 21 kd band was seen for ras in the Western blots (Figure 2a). But 43% of normal and 32% of CML samples showed an additional band at 25 kd at all the time points, indicating existence of differential isoprenylation of ras. For densitometry analysis, both the bands were considered collectively.

On fMLP stimulation, 50% normal samples showed increase in ras at early time points. With increasing time, this percentage increased to 79% resulting in a significant increase in ras (about 1.5 fold) at 30 and 45 min (Table 1a). On fMLP stimulation, CML PMNL showed very little or no change in ras expression (Figure 2a). Normal and CML PMNL expressed similar basal levels of ras. But after stimulation for 30 min, normal PMNL showed significantly higher ras levels as compared to the corresponding CML PMNL.

In FCM estimation, normal PMNL showed about two-fold increase in ras (range 1.35-4.2) after fMLP stimulation (Figure 2c). The increase was significant at 5 and 10 min of fMLP stimulation (Table 1b). In CML PMNL, the majority of the samples did not show any response to fMLP. Only at 45 min of stimulation, 36% of the samples showed increase in ras resulting in a statistically significant increase (1.2 fold) in average ras expression (Figure 2c, Table 1b).

Basal ras levels in normal PMNL were slightly higher than in CML PMNL. But an extremely delayed and low response of CML PMNL to fMLP resulted in significantly higher ras levels in normal PMNL, stimulated for 5 and 10 min than the respective CML PMNL. Hammond et al. have suggested that intracellular signalling could occur through modulation of the oscillations in response to stimulus. Cancer can result from changes in the oscillation frequencies, amplitudes and phasings of signaling molecules [13]. In *Dictyostelium discoideum *ras activation stimulates a small amount of preexisting, membrane-associated PI3K, causing F-actin polymerization [14]. Thus, defective ras dynamics might lead to defective actin polymerization. The present findings reveal that on fMLP stimulation, ras levels increased only in normal PMNL, indicating defects in signals regulating ras expression in CML PMNL.

### Intracellular localization of ras in normal and CML PMNL is comparable

Unstimulated and stimulated normal and CML PMNL showed diffused cytoplasmic staining for ras. In normal, bleb formation, observed at 0.5 min of stimulation, was associated with an increase in ras. The fluorescence intensity increased with increase in stimulation time, indicating an increase in ras. Only 50% CML PMNL that showed morphological changes on stimulation showed slight increase in ras expression. Overall change was not noted in the levels of ras in response to stimulation. There was no distinct difference in the localization of ras in normal and CML PMNL (Additional file 1: Figure S1 & Additional file 2: Figure S2). Since only 10% of the total GTPase undergoes a transition from inactive to active state, limitations of the used LCM technology and lack of specific probe could have made these alterations undetectable. Thus, at present, defects in stimulation of actin polymerization could be partly attributed to alterations in dynamics of ras expression.

The morphological pathway is branched into PI3K-dependent and PI3K-independent pathways. The PI3K-dependent pathway also depends on protein kinase C (PKC) ξ and Akt/PKB, and controls 70-80% of F-actin. PKCξ is involved in pseudopodia formation and oxidative burst [12,15]. In fMLP stimulated PMNL, translocation of PKCξ to the plasma membrane started at 1 min, but peripheral actin polymerization was observed by 30 seconds [4]. This difference in time kinetics suggests that PKCξ may not have direct role in spatial distribution of F-actin in PMNL. The PI3K-independent pathway depends on rhoGTPases, ROCK, src kinases and NADPH, and is modulated by cAMP [16]. Ras and its associated rhoGTPases - rac, rho and cdc42, play an important role in the spatial and temporal organization of actin, and regulate cell adhesion and motility. Cdc42 is required for cell polarity and rac1 for protrusive activity [17]. Basal rhoA activity is necessary to maintain cell adhesion. This pro-adherent effect of rhoA probably counterbalances the effects of rac1 and Cdc42 as rac1 must inhibit rhoA to exert its activity toward myosin [18]. Since rhoGTPases cross-activate each other, balanced control of this activation determines outcomes like cell polarization, directional motility and substrate adhesion. As mentioned earlier, CML PMNL showed defects in cell polarization, adhesion, motility, pinocytosis, etc., and it was suggested that defective actin polymerization might have contributed to these defects [19]. In CML, defective actin polymerization may result in early egress of PMNL and immature myeloid cells from the bone marrow. Therefore, to understand defective actin polymerization in CML PMNL further, expression of GTPases - rac1, and rhoA, was examined.

### Different rac1 isoforms are stimulated in normal and CML PMNL

In the Western blots 50% of normal and 59% CML samples showed a single band of rac1 at 21 kd, at all the time points studied (Figure 2a). But the remaining samples showed two bands, at 21 kd and 25 kd. The 25 kd band could be of the rac1b protein, as the molecular weight of recombinant rac1b isolated from *E. coli *is reported to be higher than 21 kd [20]. Therefore, for densitometry analysis, both the bands were considered collectively.

In Western blot studies, about 60% of normal and CML samples showed increase in rac1 levels at early time points of fMLP stimulation. Increase in rac1 expression was followed by a drop at later time points of fMLP stimulation. In normal, this increase was not significant., but in CML PMNL, increase at 5, 10 and 30 min of fMLP stimulation was statistically significant (Table 1a). Rac1 levels were comparable in normal and CML PMNL, under unstimulated and stimulated conditions. But the major responder bands in normal and CML were 25 kd and 21 kd, respectively. The 25 kd band could be of rac1b. Rac1b acts like a fast cycling GTPase [21] and induces formation of lamellipodia in NIH3T3 [22]. Similarly in normal PMNL too, rac1b could be responsible for actin polymerization in lamellipodia. Though unstimulated CML PMNL showed higher levels of total rac1, and these levels increased further in response to stimulation, lower response of rac1b might have resulted in the absence of lamellipodia in CML PMNL leading to the absence of chemotaxis.

In FCM studies, only 50% of the normal samples showed increase in rac1 (range 1.3-2.3) at early time point, specifically at 0.5 min of fMLP stimulation and then showed a second increase (Figure 2d). Of the remaining, 30% samples showed a drop and 20% samples showed delayed increase in rac1 levels. Hence, the increase in the average median channel for rac1 on stimulation was statistically insignificant (Table 1b). In CML PMNL, majority of the samples showed a drop (range 10- 60%) in rac1 levels on stimulation at early time points of stimulation followed by a partial recovery. At later time points, only 21% of the samples showed a true increase in rac1 levels (Figure 2d). Thus, CML PMNL showed a significant drop in rac1 levels after 0.5, 5, 10 and 45 min of stimulation (Table 1b). FCM studies showed higher expression of rac1 in unstimulated CML PMNL than that in normal PMNL. On fMLP stimulation, the rac1 levels increased in normal PMNL and dropped in CML PMNL. Hence, significant difference between rac1 levels of both was seen at 45 min of fMLP stimulation (Table 1b).

The differences in results, between Western blot and FCM, could be because the major responder band in the two populations was different and the antibodies used have different affinities towards these bands. Secondly, depending on the localization of the 21 kd and 25 kd rac1 proteins in the cell, in FCM, the antibody could have had altered accessibility.

### fMLP stimulated transport of rac1 to the cell membrane is negligible in CML PMNL

In unstimulated normal PMNL, rac1 expression was less in cytoplasm and more on the membrane. On stimulation, the fluorescence intensity increased, but the distribution pattern of rac1 remained same. In the majority of unstimulated CML samples rac1 was distributed everywhere. On fMLP treatment, rac1 distribution remained unaltered. In both, changes in rac1 levels seen by LCM matched with that observed by FCM, and were independent of morphological changes (Additional file 3: Figure S3 & Additional file 4: Figure S4).

The major difference in the distribution of rac1 between normal and CML PMNL was that normal PMNL showed a higher concentration of rac1 on the membrane, suggesting that CML PMNL could be defective in translocation of rac1 to the cell membrane. Alternatively, in view of the high binding of LCM antibody to the 25 kd band, the major portion of peripheral rac1 could be 25 kd suggesting higher expression of post-translationally modified rac. If this were true, then access to 21 kd would be lowered. This could lead to weak fluorescence in stimulated CML PMNL. However, unaltered rac1 distribution on stimulation indicated an absence of significant changes in rac1 localization.

### fMLP stimulated degradation of rhoA is slower in CML PMNL

In the Western blots about 50% normal and 60% CML samples showed a drop in rhoA levels at early time points of fMLP stimulation, resulting in a 20% drop in average rhoA levels. In normal, the drop gradually increased to a significant level (Figure 2a) (Table 1a). But in CML the decrease was statistically significant only at 45 min of stimulation (Table 1a).

Higher rhoA expression in unstimulated CML PMNL, as compared to that in normal PMNL, was not statistically significant. But on stimulation, differences between the rhoA levels of the two populations increased gradually, resulting in significantally lower (2-3 folds) levels in normal at later time points.

In FCM analysis of normal PMNL, at early time points of stimulation mixed response was seen. Later, a majority of the samples showed decrease in rhoA (Figure 2e, Table 1b). In CML PMNL, about 50% samples showed a drop in rhoA at early time points, but eventually showed an increase. As a result, at the later time points, rhoA levels in stimulated CML PMNL remained at par with the basal level (Table 1b).

A comparison between normal and CML PMNL showed that unstimulated normal PMNL as well as those during early stimulation have higher rhoA expression. But, at 45 min of stimulation, the picture reversed. Though the trend seen for rhoA expression was similar by Western blotting and FCM, the latter did not yield significant differences.

### Intracellular distribution of rhoA is comparable in normal and CML PMNL

In the majority of samples, unstimulated normal and CML PMNL showed cytoplasmic rhoA. In 20% normal and 40%CML samples, unstimulated PMNL showed rhoA in the peripheral region below the F-actin layer. In both, rhoA distribution remained unaltered on fMLP treatment. Changes in rhoA levels were similar to those seen using FCM, and were not associated with morphological changes (Figure 3a).

### Co-localization of F-actin with rhoA

In unstimulated and stimulated normal and CML PMNL, peripherally concentrated F-actin did not co-localize with rhoA, while most of the diffused cytoplasmic actin co-localized with rhoA (Figure 3a). This was reflected in the lack of statistically significant differences in the co-localization coefficient of unstimulated (0.63 ± 0.04) and fMLP-stimulated PMNL (0.63 ± 0.02). CML PMNL showed lower co-localization coefficients as compared to the normal (0.57 ± 0.02). Moreover, co-localization coefficients were more scattered in stimulated CML PMNL (range 0.13 - 0.86) than that in normal PMNL (range 0.29 - 0.86). Less than one values of average co-localization coefficients in normal and CML PMNL further supported the observation of lack of colocalization of major part of F-actin with rhoA. In contrast to this, in normal and CML PMNL, all rhoA was co-localized with F-actin.

Some variation was seen within the unstimulated normal population with respect to co-localization of F-actin with rhoA. To group the majority of normal samples as a tight population and to segregate samples that behaved differently from the rest, a cut off percentage (20 percentile) was applied. All the samples above the cut off were considered as "normal" and all the samples below the cut off were categorized as "non-normal". The percentage of samples behaving as non-normal was similar under unstimulated and stimulated conditions (Table 2).

**Table 2:** Distribution of samples according to co-localization of F-actin with rhoA

fMLP Stimulation	PMNL	Sample Distribution	
		
		Non-normal	Normal
Unstimulated	Normal	14%	86%
	
	CML	32%	68%

0.5 min	Normal	14%	86%
	
	CML	***45%***	55%

5 min	Normal	14%	86%
	
	CML	32%	68%

10 min	Normal	21%	79%
	
	CML	36%	64%

30 min	Normal	14%	86%
	
	CML	***55%***	45%

45 min	Normal	21%	79%
	
	CML	18%	82%

20% percentile is used as a cut off for defining the pattern of co-localization of F-actin with rhoA in normal and CML PMNL, as "normal" and "non-normal". Values in bold and italics mark the increased "non-normal" population.

To segregate CML samples from the normal samples, the same cut off was applied to the CML PMNL. In CML, under unstimulated conditions, 32% of the samples behaved as non-normal. On stimulation, the percentage of non-normal samples increased to 45% and to 55% at 0.5 min and 30 min of fMLP stimulation, respectively (Table 2). Thus, 0.5 and 30 min of fMLP stimulation appeared to be critical to differentiate between normal and CML PMNL.

### Ras and rhoA are critical GTPases in normal and CML PMNL, respectively

GTPases play a key role in signal transduction, leading to spatial and temporal organization of cytoskeleton proteins, especially actin. In order to understand the signalling network of GTPases better and to see if the change in expression of one GTPase had any correlation with change in correlation of other GTPase or with F-actin, bivariate correlation analysis was used. This analysis enables to measure the strength of linear relationship between variables. To further understand if the correlated variables as determined by the bivariate correlation were directly linked or whether they were indirectly linked, partial correlation analysis was done. It was also used to check if correlation between non-correlated variables as determined by bivariate correlation was masked due to other variables. Bivariate and partial correlation analysis of the data indicated negative correlation between G-actin and F-actin at 30 min of fMLP stimulation in normal PMNL. Moreover, ras emerged as the critical GTPase regulating expression of the rhoGTPases - rhoA and rac1, and also of G-actin and F-actin (Figure 3b). In CML PMNL, rhoA took a central place in the GTPases involved in actin polymerization instead of ras. In CML PMNL, constitutively active tyrosine kinase, bcr-abl might be independently activating ras, rhoA and rac1, even in the absence of an external stimulus like fMLP. This probably resulted in absence of ras control on the actin polymerization pathway and established new direct links like rac1 - rhoA, F-actin - rhoA and F-actin - ras (Figure 3b). RhoA also showed a suppression effect. This altered regulation of GTPase- rhoA might have led to deregulated actin polymerization and thereby defects in various actin-dependent functional events.

Besides morphology or motility pathway, dynamics of actin plays crucial role in cell division. Hence, if our conclusion of crucial role of rhoA in CML pathogenesis is true then it should be reflected in proliferation of CML cells. Since PMNL used in these studies are terminally differentiated cells, effect of rhoA on cell proliferation cannot be tested in these cells. But it needs to be tested either in CML cell lines or mononuclear cells from bone marrow of CML patients. Resistance to imatinib, a bcr-abl tyrosine kinase inhibitor which is the first line of CML chemotherapy is the major challenge for CML in clinics. Hence, we have used imatinib sensitive and resistant CML cell lines to validate our conclusion derived from the above-mentioned studies in CML PMNL.

K562 is a pluoripotent CML cell lines derived from CML patient in blastic crisis and is sensitive to imatinib. Another cell line chosen was BaF3/bcr-abl/T315I that expresses the most common and most resistant bcr-abl mutant. To test specificity of the hypothesis, HL-60, a bcr-abl negative promyelocytic leukemic cell line was used as a control. Since activation of rhoGTPases is important for their functioning, activation of rhoA was inhibited by using C3 exoenzyme from *Clostridium botulinum*, a known specific inhibitor of rhoA activation. To test involvement of signalling molecules downstream of rhoA, Y27632 - an inhibitor of ROCKI kinase was used. At transcription level, rhoA was targeted by using validated antisense oligonuleotides (ASODN).

**Figure 4:**
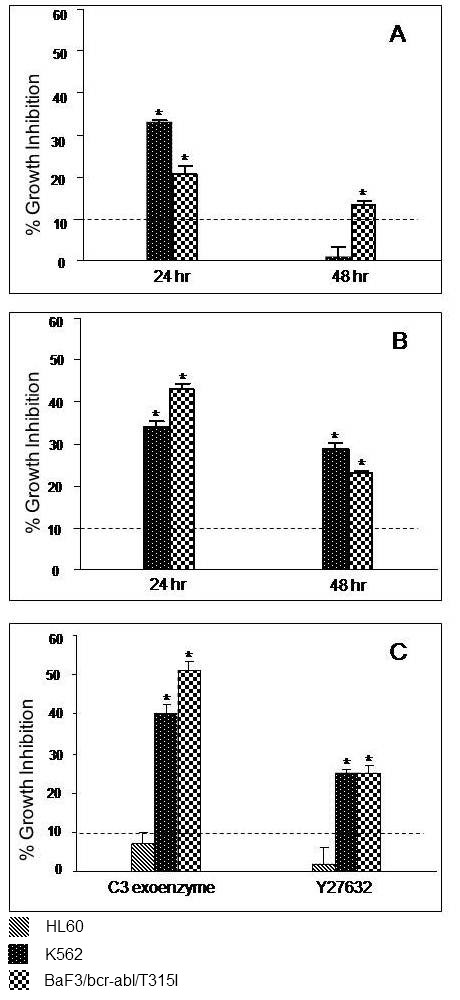
**Effect of rhoA and ROCK inhibition on cell growth**: Percent growth inhibition in HL60, K562 and BaF3/bcr-abl/T315I treated with - **A**) PO against rhoA, **B**) PS against rhoA, for 24 and 48 hours and **C**) C3 exoenzyme and Y-27632, for 48 hours. * Indicates significantly higher growth inhibition than the respective untreated cells and treated HL-60.

Inhibition of rhoA at the transcriptional level by using phosphodiester ASODN (PO) and phosphorothioate ASODN (PS) resulted in about 20 - 40% growth inhibition in K562 and BaF3/bcr-abl/T315I. Inhibitory effect of PO decreased by 48 hours while, effect of PS was comparatively long lasting. This differential effect could be explained on the basis of longer half-life and higher binding affinities of PS than PO. Though this inhibitory effect on cell proliferation proves our hypothesis that rhoA plays important role in CML pathogenesis, role of activation of rhoA and subsequent signalling events remains to be elucidated. When activation of rhoA and downstream signalling via ROCK were inhibited by treatment of cell lines with C3 exoenzyme and Y-27632, respectively both resulted in distinct growth inhibition in K562 and BaF3/bcr-abl/T315I, but not in HL-60 (Figure 4). Inhibition of K562 and BaF3/bcr-abl/T315I by C3 exoenzyme and Y-27632 was significantly higher than the solvent control and HL-60, suggesting that rhoA pathway inhibitors specifically inhibited growth of bcr-abl expressing cells. Moreover, higher inhibition of imatinib resistant BaF3/bcr-abl/T315I than K562 by C3 exoenzyme suggested that rhoA could be a good therapeutic target in CML.

## Discussion

To understand defective actin polymerization in CML, studies were focused on the downstream signalling molecules in the actin polymerization pathway. The present study has clearly noted higher expression of rhoA and rac1 in unstimulated and fMLP stimulated CML PMNL. Higher expression of GTPases is likely to be associated with higher expression of their active forms [23]. On stimulation, the drop in rhoA was lower in CML than that in normal. Isoprenoid substrates are essential to post-translationally modify ras and rhoGTPases [24]. Reduction of isoprenoid substrates induced up-regulation of ras, rap1a, rhoA and rhoB due to increased mRNA and protein synthesis, and decreased protein degradation. A crisis in the isoprenoid substrate levels probably decreases protein degradation [24]. In view of this, reduced degradation of rhoA in stimulated CML PMNL could be due to reduced levels of the isoprenoid substrate. The presence of high levels of rhoA results in disruption of the actin cytoskeleton and microtubules [25]. Lowered number of microtubules [26] and F-actin [4,27] have been reported in CML PMNL. Thus, consistently high levels of rhoA in CML PMNL could explain the defects in cytoskeleton, cell polarization and chemotaxis.

In normal PMNL, fMLP treatment led to a decrease in rhoA and total actin levels and increase in ras and rac1b levels. These resulted in actin polymerization, formation of lamellipodia and subsequently in chemotaxis, phagocytosis, etc. In CML, kinetics of expression of ras, rac and rhoA is altered. Since rac1 and rhoA regulate each other, and rac1 must inhibit rhoA to exert its motility related effects, it can be speculated that altered dynamics of these GTPases in CML could result in defective actin polymerization and subsequent actin-dependent functions. Further studies on expression of active-rhoGTPases may elucidate more distinctly, the differences in normal and leukemic populations.

CML PMNL remain in circulation for a longer period than the corresponding normal PMNL. Mechanism of this longevity is not clearly understood. GTPases also act as molecular switches controlling proliferation and differentiation of cells [28,29]. Over expression or mutation can make GTPases constitutively active, and may result in dysregulated cell signalling and proliferation [23]. Ras along with rac1 or rho has been implicated in tumorigenesis [30] and cell transformation [31,32]. Aberrant activation of rhoGTPases per se promotes uncontrolled proliferation, invasion and metastatic properties of tumor cells [33]. Over expression of rhoGTPases has been reported in many cancers [34-38]. Alteration in rhoA levels has been correlated with malignancy [39]. It is suggested that cancer is associated with higher expression, rather than any mutations, of rhoGTPases [29,37].

Rac1 and rho regulate cell cycle progression through the G1 phase [40], and also regulate the expression of growth-promoting genes like c-fos that are required for cell cycle progression [41]. Significantly increased rac1 RNA and protein has been reported in patients with aggressive breast cancer and oral squamous cell carcinoma [20,42]. Over-expression of rac1 induces a strong proliferative response in NIH3T3 [43]. Rac1 stimulates transcription of cyclin D1 via PAK, thereby promoting cell cycle progression through G1 [40]. RhoA facilitates entry into S phase by degradation of the cyclin-dependent kinase inhibitor p^27kip1 ^[44]. Additionally, constitutively activated mutants of the rhoGTPases stimulate DNA synthesis leading to cell cycle progression. Hence, higher expression of rac1 and rhoA in CML PMNL could be responsible for increased proliferation of these cells.

In normal PMNL, ras appeared to be a critical GTPase regulating the expression of other GTPases and actin. But in CML PMNL, rhoA emerged as the critical GTPase. This altered behavior of rhoA could be responsible for the diseased state. Since the chimeric bcr-abl gene has Dbl homology (DH) domain and wild type bcr has racGAP domain, role of rac pathway in leukemogenesis was predicted and tested. For example, the oncogenic tyrosine kinase bcr-abl has been shown to activate rac via vav and active rac was shown to be important in leukemogenesis [45]. DH domain of bcr-abl activates NF-kB via P38 MAPK activation [46]. Up regulation of rhoA gene and rhoA regulator LARG is reported in bcr-abl expressing cell lines [47,48]. Sahay et al. have shown that p210 bcr-abl can stimulate rhoA activation independent of its tyrosine kinase activity via its DH domain and inhibition of rhoGEF activity resulted in impairment of transforming activity of p210 that is measured by anchorage independent growth [49]. Role of rhoA in amoeboid motility of p210 bcr-abl expressing cells was demonstrated by Daubon et al. [50]. Unwin et al. have shown effects of inhibitors of rho on growth and migration of bcr-abl positive cells [47,51]. Burthem et al have shown that ROCK inhibitors Y-27632 and fasudil selectively inhibited growth of CD34 positive CML progenitor cells [52]. Recently, role of rhoGTPases in heamtopoiesis and haemopathies including CML is well reviewed by Mulloy et al [53]. But to our knowledge, there are no reports in the clinical samples, collectively illustrating expression of ras and rhoGTPases and correlating to the structural event like polymerization of actin. In the present report, effects of stimulation on expression of these molecules are also studied. Probably, this is the first report wherein rhoGTPase pathway is systematically dissected in clinical samples, using various experimental approaches and over-expression of rhoA is reported in CML. In collective analysis of these parameters, a statistically significant model has emerged suggesting that rhoA is the critical GTPase regulating expression of other GTPases and actin in CML PMNL. Specific growth inhibition of bcr-abl expressing cells by rhoA and ROCK inhibitors and more inhibition of imatinib resistant cell line by rhoA activation inhibitor than ROCK inhibitor observed by us, supported our hypothesis and identified rhoA as a potential target for the development of therapeutic drugs. Ohmine et al. have shown by microarray analyses that rhoA and ras p21 protein activator (RASAP) levels are increased in imatinib resistant KCL22 cell line [47]. These observations explain the higher growth inhibition of BaF3/bcr-abl/T315I than K562.

Often, the regulatory molecules are predicted by microarray studies on the basis of transcriptional up regulation. However, the transcriptional changes need not be always reflected at the translational levels. Proteomics is another tool that is used to identify regulatory molecules. Our approach of dissecting defective signalling pathway at the molecular and cellular level is a kind of focused proteomics and cellomics. Hence, in comparison to the predictions on the basis of microarray or proteomics alone, hypothesis based on the statistical analysis of the expression of the signalling molecules per se in clinical samples is more likely to be translated successfully in clinics.

## Conclusions

To summarize, in CML PMNL expression and spatial organization of GTPases - ras, rhoA and rac has altered, probably leading to altered actin dynamics. Hence, the altered actin dependent functions in PMNL could be a result of altered GTPases. In correlation analysis, rhoA has emerged as the key regulator in CML. Hence it was hypothesized that rhoA is the crucial factor regulating altered behaviour of CML cells. This hypothesis was validated by studying effect of rho/ROCK pathway inhibitors on imatinib sensitive and resistant CML cell lines. In view of these results, rhoA is proposed as a therapeutic target for CML.

## Materials and methods

### Reagents

Antibodies and kits were obtained from various sources listed here: Anti-actin, anti-rac1, alkaline phosphatase conjugated anti-rat antibody and anti-rhoA [Santacruz Inc., Santacruz, CA, USA]; anti-H-ras [Oncogene, San Diego, CA, USA]; enhanced chemiluminescence kit containing alkaline phosphatase conjugated goat anti-rabbit antibody (GAR-AP) [New England Biolabs Inc., Ipswich, MA, USA]; Alexa 488-conjugated goat anti-mouse antibody (GAM) [Molecular Probes, Carlsbad, CA, USA]; FITC conjugated anti-ras and TRITC conjugated anti-rac1 [Becton Dickinson, Franklin Lakes, NJ, USA]; goat anti-mouse antibody alkaline phosphatase conjugated (GAM-AP) [Sigma, St. Louis, MO, USA] and cell counting kit-8 (CCK-8) [Dojindo Laboratories, Kumamoto, Japan].

### Clinical samples and cell lines

After taking written consent, peripheral blood was collected from healthy volunteers (control) and CML patients in chronic phase; before commencement of therapy and processed simultaneously. PMNL were isolated on a ficoll-hypaque gradient [54] and immediately used for the experiments.

Bcr-abl expressing cell lines K562 and BaF3/bcr-abl/T315I were used along with bcr-abl negative cell line HL-60, as a control. The cell lines were maintained in RPMI 1640 containing 1X AB-AM and 10% (v/v) fetal bovine serum, at 37°C in a humidified atmosphere containing 5% CO_2_.

### PMNL stimulation

PMNL were stimulated at 37°C with 10^-8^M fMLP for various time durations. For Western blotting, cells were quickly pelleted at 4°C and lysed in Laemmli's sample buffer containing CompleteTM protease inhibitor [Roche, Mannheim, Germany]. For flow cytometric and microscopic analysis, cells were fixed with paraformaldehyde. Unstimulated PMNL were used as control.

### Western blotting

Lysates form one million cells were loaded on 16% SDS-PAGE. After confirming equal protein loading by staining the blots, actin and GTPases were detected. Protein band density was quantitated as mean area density, using the software-Labwork.

### Immunofluorescent staining

Fixed PMNL were permeabilized with Triton-X100 and stained using FITC-anti-ras or TRITC-anti-rac or anti-rho followed by Alexa 488-GAM. For F-actin staining, TRITC-phalloidin was used [4]. The cells were analysed by FCM using FACScalibur (Becton-Dickinson) and LCM MRC1024 (Biorad) using Lasersharp software. A minimum of 10,000 cells were analysed by FCM. The median channel number was taken as a measure of fluorescence intensity. To precisely study localization of the molecules, Z-series images of 1 μm thickness were analysed by LCM.

### Growth inhibition assay

Cell lines were treated with inhibitor of rhoA GTPase activation - C3 exoenzyme (10 μg/ml), ROCK I inhibitor - Y-27632 (10 μM) and ASODN targeted against rhoA (1 μM). Cell proliferation was monitored at 24 and 48 hrs., by using CCK-8 assay [55]. Effect of treatment on cell growth was expressed as percent growth inhibition.

### Statistical analysis

Wilcoxon signed rank test was used to compare the values within normal and CML samples. Mann-Whitney test was used to compare normal with CML and K562 and BaF3/bcr-abl/T315I with HL-60. Bivariate and partial correlation analyses were used to study the relationship between all the parameters. *p *< 0.05 was considered to be statistically significant.

## Abbreviations

CCK-8: Cell counting kit-8; CML: chronic myeloid leukemia; FCM: flow cytometry; fMLP: n-formyl-Methionyl-Leucyl-Phenyl alanine; LCM: laser scanning confocal microscope; PKC: protein kinase C; PMNL: Polymorphonuclear leucocytes.

## Competing interests

The authors declare that they have no competing interests.

## Authors' contributions

N.R.N. conceived and designed the experiments. P.R.M. did experiments with clinical samples. S.H.A. was a clinical collaborator who identified the patients and provided clinical samples. M.B.P. did growth inhibition assay using cell lines. P.R.M and M.B.P. analyzed the respective data under guidance of N.R.N. P.R.M. and N.R.N. wrote the paper.

## Supplementary Material

Additional file 1 **Figure S1. H-ras localization in normal PMNL**: Middle Z planes and 3D LCM images showing distribution of H-ras in unstimulated and fMLP stimulated (0.5, 5, 10, 30, 45 min) normal PMNL. The central panel shows the corresponding transmission images of the fluorescent images. For studying distribution of GTPases, 'thermo1' LUT based on the fluorescence intensity was applied to all the fluorescence images in this figure. Fluorescence intensity reflects the expression level of the protein of interest. The pseudocolour code used was as follows: Dark blue: extremely low level of expression; Light blue: low level of expression; Yellow: moderate level of expression; Orange: high level of expression; Red: extremely high level of expression. Click here for file

Additional file 2 **Figure S2. H-ras localization in CML PMNL**: Middle Z planes and 3D LCM images showing distribution of H-ras in unstimulated and fMLP stimulated (0.5, 5, 10, 30, 45 min) CML PMNL. The central panel shows the corresponding transmission images of the fluorescent images. For studying distribution of GTPases, 'thermo1' LUT based on the fluorescence intensity was applied to all the fluorescence images in this figure. Fluorescence intensity reflects the expression level of the protein of interest. The pseudocolour code used was as follows: Dark blue: extremely low level of expression; Light blue: low level of expression; Yellow: moderate level of expression; Orange: high level of expression; Red: extremely high level of expression. Click here for file

Additional file 3 **Figure S3. Rac1 localization in normal PMNL**: Middle Z planes and 3D LCM images showing distribution of rac1 in unstimulated and fMLP stimulated (0.5, 5, 10, 30, 45 min) normal PMNL. The central panel shows the corresponding transmission images of the fluorescent images. For studying distribution of GTPases, 'thermo1' LUT based on the fluorescence intensity was applied to all the fluorescence images in this figure. Fluorescence intensity reflects the expression level of the protein of interest. The pseudocolour code used was as follows: Dark blue: extremely low level of expression; Light blue: low level of expression; Yellow: moderate level of expression; Orange: high level of expression; Red: extremely high level of expression. Click here for file

Additional file 4 **Figure S4. Rac1 localization in CML PMNL**: Middle Z planes and 3D LCM images showing distribution of rac1 in unstimulated and fMLP stimulated (0.5, 5, 10, 30, 45 min) CML PMNL. The central panel shows the corresponding transmission images of the fluorescent images. For studying distribution of GTPases, 'thermo1' LUT based on the fluorescence intensity was applied to all the fluorescence images in this figure. Fluorescence intensity reflects the expression level of the protein of interest. The pseudocolour code used was as follows: Dark blue: extremely low level of expression; Light blue: low level of expression; Yellow: moderate level of expression; Orange: high level of expression; Red: extremely high level of expression. Click here for file
