# Supplementary figures and images for: RhoA: A therapeutic target for chronic myeloid leukemia
Source: Mol Cancer. 2012 Mar 25;11:16. doi: 10.1186/1476-4598-11-16 (PMC3353160; doi:10.1186/1476-4598-11-16)

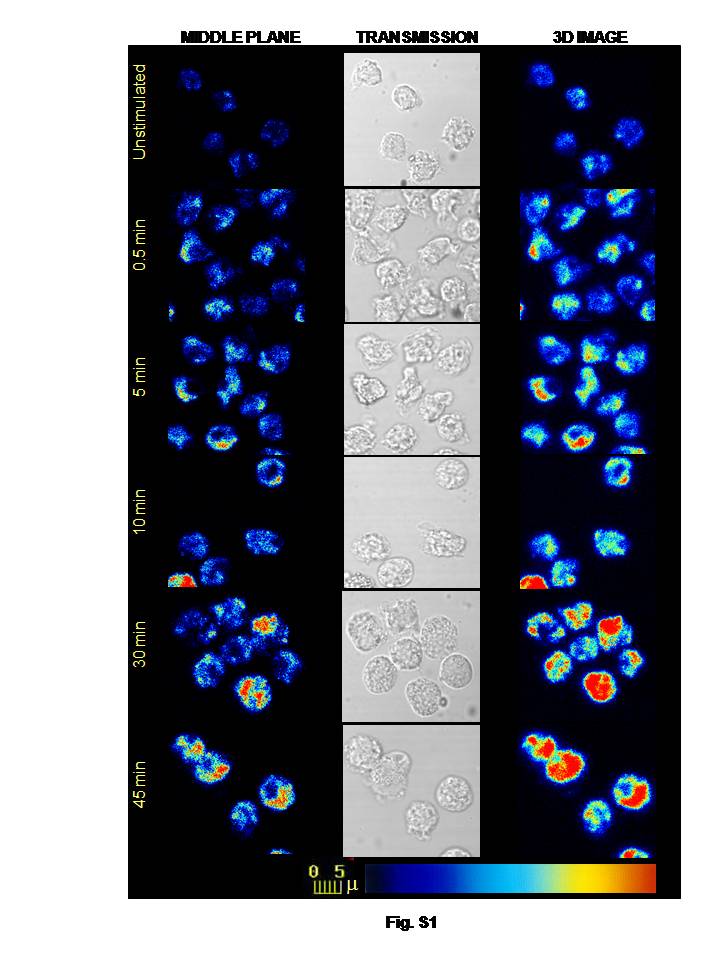

Supplement: Additional file 1 — Figure S1. H-ras localization in normal PMNL: Middle Z planes and 3D LCM images showing distribution of H-ras in unstimulated and fMLP stimulated (0.5, 5, 10, 30, 45 min) normal PMNL. The central panel shows the corresponding transmission images of the fluorescent images. For studying distribution of GTPases, 'thermo1' LUT based on the fluorescence intensity was applied to all the fluorescence images in this figure. Fluorescence intensity reflects the expression level of the protein of interest. The pseudocolour code used was as follows: Dark blue: extremely low level of expression; Light blue: low level of expression; Yellow: moderate level of expression; Orange: high level of expression; Red: extremely high level of expression. [file 1476-4598-11-16-S1.JPEG]

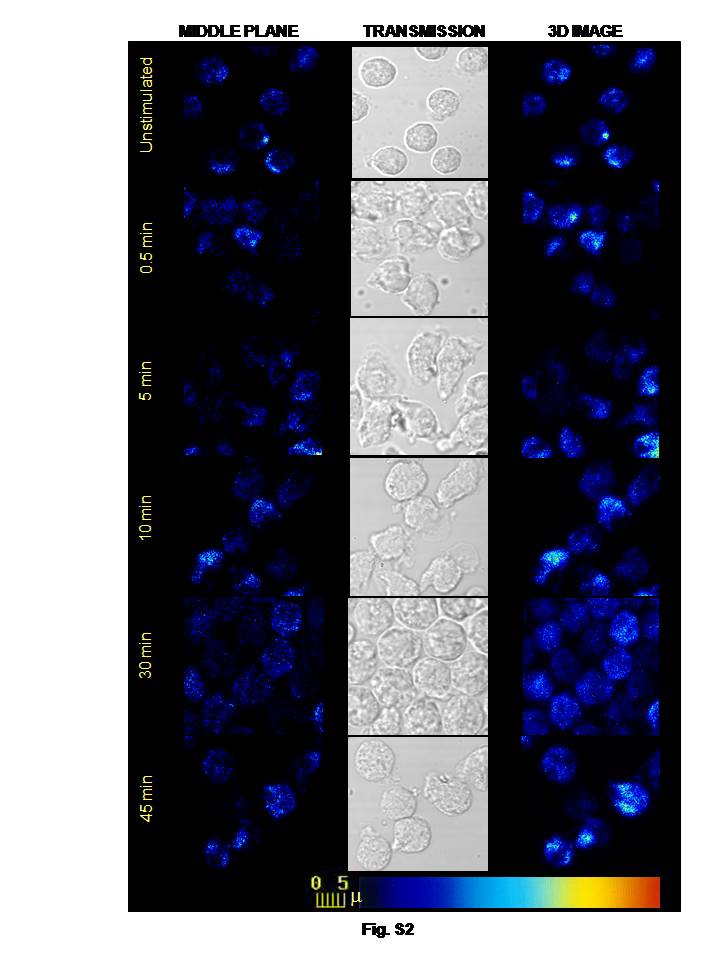

Supplement: Additional file 2 — Figure S2. H-ras localization in CML PMNL: Middle Z planes and 3D LCM images showing distribution of H-ras in unstimulated and fMLP stimulated (0.5, 5, 10, 30, 45 min) CML PMNL. The central panel shows the corresponding transmission images of the fluorescent images. For studying distribution of GTPases, 'thermo1' LUT based on the fluorescence intensity was applied to all the fluorescence images in this figure. Fluorescence intensity reflects the expression level of the protein of interest. The pseudocolour code used was as follows: Dark blue: extremely low level of expression; Light blue: low level of expression; Yellow: moderate level of expression; Orange: high level of expression; Red: extremely high level of expression. [file 1476-4598-11-16-S2.JPEG]

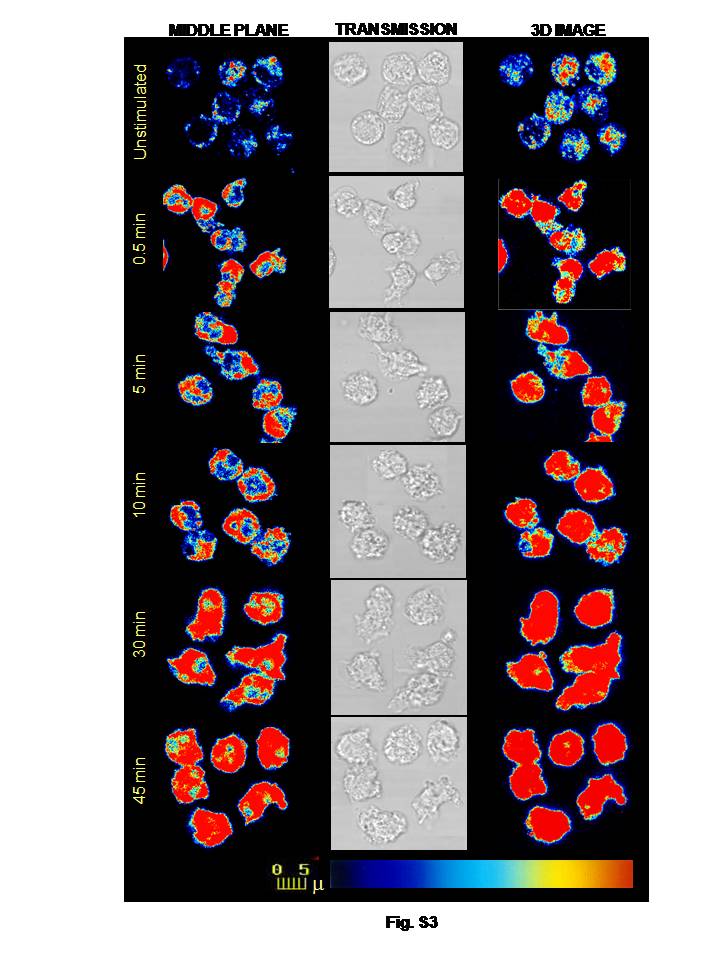

Supplement: Additional file 3 — Figure S3. Rac1 localization in normal PMNL: Middle Z planes and 3D LCM images showing distribution of rac1 in unstimulated and fMLP stimulated (0.5, 5, 10, 30, 45 min) normal PMNL. The central panel shows the corresponding transmission images of the fluorescent images. For studying distribution of GTPases, 'thermo1' LUT based on the fluorescence intensity was applied to all the fluorescence images in this figure. Fluorescence intensity reflects the expression level of the protein of interest. The pseudocolour code used was as follows: Dark blue: extremely low level of expression; Light blue: low level of expression; Yellow: moderate level of expression; Orange: high level of expression; Red: extremely high level of expression. [file 1476-4598-11-16-S3.JPEG]

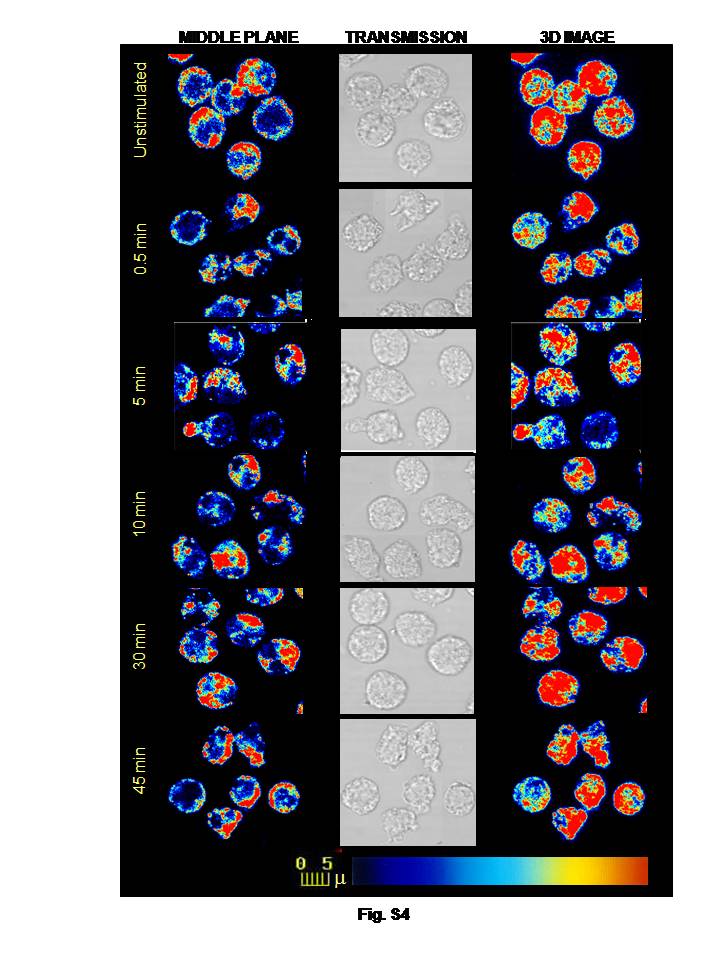

Supplement: Additional file 4 — Figure S4. Rac1 localization in CML PMNL: Middle Z planes and 3D LCM images showing distribution of rac1 in unstimulated and fMLP stimulated (0.5, 5, 10, 30, 45 min) CML PMNL. The central panel shows the corresponding transmission images of the fluorescent images. For studying distribution of GTPases, 'thermo1' LUT based on the fluorescence intensity was applied to all the fluorescence images in this figure. Fluorescence intensity reflects the expression level of the protein of interest. The pseudocolour code used was as follows: Dark blue: extremely low level of expression; Light blue: low level of expression; Yellow: moderate level of expression; Orange: high level of expression; Red: extremely high level of expression. [file 1476-4598-11-16-S4.JPEG]
